# Exploring the immune enhancement effect of radiotherapy combined with NK cells on non-small cell lung cancer based on proteomics technology

**DOI:** 10.3389/or.2026.1853377

**Published:** 2026-10-01

**Authors:** Cui Chang, Tang Youming, Lu Ying, Lei Ningning, Guo Chunhui, Wei Dekai, Liang Mei, Wu Qiyuan, Liu Gechen, Wu Fasheng, Chen Jibing

**Affiliations:** 1 Graduate School, Guangxi University of Chinese Medicine, Nanning, Guangxi, China; 2 The Second Affiliated Hospital of Guangxi University of Chinese Medicine, Nanning, Guangxi, China; 3 The Second School of Clinical Medicine, Guangdong Medical University, Dongguan, Guangdong, China

**Keywords:** complement system, immunotherapy, NK cells, non-small cell lung cancer, proteomics, radiotherapy, tumor microenvironment

## Abstract

**Background:**

Although radiotherapy (RT) and natural killer (NK) cell-based therapy have shown promising potential in the treatment of non-small cell lung cancer (NSCLC), the therapeutic efficacy and safety of their combination remain to be fully elucidated. In this study, we evaluated the efficacy and safety of combined RT and NK cell therapy and performed preliminary proteomic profiling to characterize its potential effects on the tumor immune microenvironment (TIME), providing new insights and a theoretical and clinical basis for the development of NK cell-based therapeutic strategies for NSCLC.

**Methods:**

C57BL/6-derived subcutaneous Lewis lung carcinoma models were established to determine the minimal effective dose of both radiotherapy and adoptively transferred natural killer cells. Based on the MED-derived findings, the mice were further randomized into five experimental groups for therapeutic efficacy evaluation: the Control group, the RT group, the NK group, the combination therapy group (RN group), and the repeated combination therapy group (RN2 group). Tumour volume and body weight were monitored longitudinally. Differentially abundant proteins were interrogated by Gene Ontology enrichment and Kyoto Encyclopaedia of Genes and Genomes (KEGG) pathway analyses to systematically describe the synergistic antitumor efficacy of differentially expressed proteins.

**Results:**

Compared with RT or NK cell-based immunotherapy as monotherapies, the RN group exhibited markedly enhanced tumor growth inhibition, with no overt treatment-related toxicities observed. Following the second cycle of combined therapy, mice in the RN2 group displayed a more pronounced reduction in tumor growth rate with those in the single-cycle combination cohort; notably, no significant adverse events associated with the combined regimen were detected throughout the entire experimental duration. Proteomic profiling unraveled that RT disrupted the core regulatory networks governing intracellular DNA replication and repair processes in tumor cells in a therapy-specific manner, whereas NK cell immunotherapy potently and selectively activated the classical pathway of the host complement system.

**Conclusion:**

Collectively, RT and NK cell therapy demonstrate superior synergistic efficacy and no overt toxicity was observed, with repeated treatments further delaying tumor progression. Mechanistically, proteomics shows that RT disrupts DNA replication/repair networks, whereas NK cell therapy was associated with upregulation of multiple complement components, including those belonging to the classical and lectin pathways, suggesting a potential involvement of these pathways.

## 1 Introduction

Lung cancer remains the leading cause of cancer-related mortality worldwide, with NSCLC accounting for the vast majority of cases (1, 2). Currently, surgical resection constitutes the standard of care for early-stage NSCLC, whereas therapeutic options for unresectable locally advanced or metastatic disease are predominantly limited to chemotherapy, RT, and targeted therapy, among others (3). Notably, RT plays a pivotal role in the management of oligometastatic disease or palliative care settings (4). Beyond its direct cytotoxic effects on tumor cells, RT engages in profound crosstalk with both the innate and adaptive immune systems (5). Specifically, RT not only reshapes the TIME by eliminating sensitive cells and activating resistant populations but also induces immunogenic cell death in tumor cells (6). The tumor-associated antigens (TAAs) and damage-associated molecular patterns (DAMPs) released during this process—including high-mobility group box 1 (HMGB1), heat shock proteins (HSPs), and adenosine triphosphate (ATP)—can enhance the antigen-presenting capacity of dendritic cells (DCs), thereby further orchestrating T cell-mediated specific antitumor immunity (7).

In recent years, immune checkpoint inhibitors targeting the programmed cell death protein-1 programmed cell death protein-1/Programmed Death-Ligand 1 (PD-1/PD-L1) pathway have improved the prognosis of a subset of patients with advanced NSCLC (8). However, primary or acquired resistance has constrained their overall therapeutic efficacy (7). Thus, exploring combination therapeutic strategies with distinct mechanisms of action is of considerable clinical significance. As key effector cells of innate immunity, NK cells can directly recognize and eliminate tumor cells in an antigen-sensitization-independent manner, emerging as a promising avenue in cellular immunotherapy (9, 10). Accumulating evidence has demonstrated that NK cells are rapidly activated and initiate cytotoxic attacks against stressed, senescent, virus-infected, and cancerous cells, without the requirement for prior antigen presentation by major histocompatibility complex class I (MHC-I) molecules (11). Unlike T and B cells that express antigen-specific activating receptors, NK cells coexpress a repertoire of activating and inhibitory receptors. Upon interaction with their cognate ligands on target cells, a complex balance is established between multiple activating and inhibitory signals, with inhibitory signals predominating (11). Among NK cell receptors, CD16 is one of the most potent activating receptors; uniquely, it can either trigger signals independently or form homodimers with the adaptor molecules CD3ζ or FcRγ to transduce robust activating signals for antibody-dependent cellular cytotoxicity (ADCC)—a process by which NK cells eliminate antibody-opsonized target cells (11). Studies have shown that RT can enhance NK cell function through multiple mechanisms, including upregulating the expression of Natural Killer Group 2, Member D (NKG2D) ligands on tumor cell surfaces and promoting NK cell tumor infiltration (7, 12–14). These findings suggest that RT and NK cell therapy possess inherent mechanistic synergistic potential.

NK cells are innate immune effectors capable of rapidly eliminating tumor cells without prior antigen-specific sensitization, whereas RT induces tumor cell death while modulating TIME (15–17). RT-induced tumor damage can enhance NK-cell activation and recognition of residual malignant cells, including heterogeneous or relatively radioresistant tumor populations, thereby complementing the limitations of RT monotherapy. However, radiation-induced endogenous immune activation is often insufficient and transient, and immunosuppressive signals within the TIME may further limit its efficacy. Thus, adoptive NK-cell therapy combined with RT may enhance antitumor immunity by integrating radiation-induced immune modulation with the potent cytotoxicity of NK cells. Based on this rationale, we investigated the efficacy and safety of RT combined with NK-cell therapy in NSCLC and preliminarily explored its potential mechanisms and effects on the TIME, with the aim of providing a theoretical basis for the clinical translation of this combination strategy.

## 2 Materials and methods

### 2.1 Tumor cell culture

The mouse LLC cell line was obtained from Servicebio (Wuhan, China). LLC cells were cultured in high-glucose Dulbecco’s modified eagle medium (DMEM) complete medium (DMEM basic, Thermo Fisher Scientific, China) supplemented with 10% Fetal bovine serum (FBS, Servicebio, China) and 1% Penicillin-Streptomycin (Servicebio, China), and maintained in a humidified incubator at 37 °C with 5% CO_2_. When cell confluency reached 95%, cells were trypsinized with 0.25% Trypsin-EDTA (Servicebio, China) for subculture. During the logarithmic growth phase, cells were harvested by trypsinization, resuspended in phosphate-buffered saline (PBS, Servicebio, China), counted, and adjusted to a final concentration of 1 × 10^8^ cells/mL. Each mouse was subcutaneously inoculated with 0.1 mL of the cell suspension, corresponding to 1 × 10^7^ cells per animal.

### 2.2 NK cells expansion and flow cytometry

In the present study, human peripheral blood mononuclear cells (PBMCs) were isolated from healthy donors. NK cells were isolated, cultured, and expanded for 18 days using a serum-free NK cell culture kit (Yocon, China), in accordance with the manufacturer’s instructions. Peripheral blood was aliquoted into 50-mL tubes and centrifuged at 700 × g for 15 min at RT. The plasma layer was carefully collected, avoiding the buffy coat, and heat-inactivated at 56 °C for 30 min. After centrifugation at 900 × g for 10 min, the supernatant was subjected to a freeze–thaw cycle (−20 °C for 15 min, followed by RT thawing) and re-centrifuged at 900 × g for 10 min. The final supernatant was stored at 4 °C until use. The erythrocyte fraction obtained from the previous plasma separation step was diluted with an equal volume of normal saline and gently mixed. The diluted blood was carefully layered onto lymphocyte separation medium in fresh 50-mL centrifuge tubes at a 1:1 ratio, ensuring a clear interface between the blood and the separation medium. The tubes were centrifuged at 700 × g for 30 min at room temperature without brake. Following centrifugation, the whitish, cloudy buffy coat layer containing PBMCs was carefully aspirated and transferred to a new 50-mL tube. The cells were washed with an equal volume of normal saline and centrifuged at 700 × g for 10 min at room temperature. The supernatant was discarded, and the pellet was resuspended in 40 mL of normal saline, followed by centrifugation at 200 × g for 10 min to remove platelet contamination. After discarding the supernatant, the PBMC pellet was resuspended in pre-warmed (37 °C) complete culture medium.

T75 flasks were coated with DPBS containing YC00A at 37 °C for 2 h, washed once, and seeded with PBMCs at 2 × 10^6^ cells/mL in activation medium containing YC00B and 10% autologous plasma (total volume 30 mL). On day 3, 30 mL of activation medium with 5% autologous plasma was added. On day 5, 140 mL of activation medium with 5% autologous plasma was added, and the culture was split into two T175 flasks (total 200 mL). On day 7, when cell density exceeded 1 × 10^6^/mL, the culture was expanded 1:2 with expansion medium containing 1% autologous plasma (total 600 mL) and transferred to FEP culture bags. On days 9 and 11, additional expansion medium was added to reach final volumes of 1,200 mL and 2200 mL, respectively. Cells were harvested on day 18 for downstream applications. Finally, the expanded NK cells were harvested and cryopreserved in CRD10 serum-free cell cryopreservation medium (CRGEN, China) for future experimental use.

To characterize the phenotype of expanded cells, flow cytometry was employed to detect the NK cell-specific markers CD56 and CD16, with concurrent analysis of the T cell marker CD3 for phenotypic discrimination (18). Cryopreserved NK cells were thawed, washed, and resuspended at 1 × 10^7^ cells/mL in PBS. Aliquots of 100 µL (1 × 10^6^ cells) were stained with fluorochrome-conjugated anti-human CD3, CD16, and CD56 antibodies (5 µL each; BioLegend) after Fc receptor blockade, along with isotype and unstained controls. Following 30 min incubation at RT in the dark, cells were washed, resuspended in PBS, and acquired on a CytoFLEX flow cytometer (Beckman Coulter, United States).

For functional activity evaluation, flow cytometry was utilized to determine the expression levels of CD107a, granzyme B, and perforin in NK cells following co-culture with K562 cells (18). Expanded NK cells were co-cultured with K562 target cells at a 3:1 ratio (6 × 10^5^ NK cells: 2 × 10^5^ K562 cells) in 96-well plates for 1 h at 37 °C in the presence of anti-CD107a antibody. Protein transport inhibitor was added to the positive control group. After incubation, cells were stained with Zombie NIR for viability, followed by surface staining with anti-CD3, CD16, and CD56 antibodies. Intracellular staining for granzyme B and perforin was performed after fixation and permeabilization using BD Fix/Perm buffer. Samples were acquired on a CytoFLEX flow cytometer, and NK cells were identified as CD3^−^CD16^+^CD56^+^. The percentages of CD107a^+^, granzyme B^+^, and perforin^+^ cells within the NK cell gate were determined. A minimum of 10,000 NK cell events were collected per sample.

Target Jurkat cells were labeled with CFSE and co-cultured with NK effector cells at E:T ratios of 5:1, 10:1, and 20:1 for 4 h in a 6-well plate (2 × 10^5^ target cells per well). Target cells alone and target cells with 0.1% Triton X-100 served as spontaneous lysis and maximum lysis controls. After incubation, cells were harvested and stained with Annexin V and PI in binding buffer for 15 min at room temperature in the dark. CFSE^+^ target cells were gated by flow cytometry, and the percentages of Annexin V^+^ and/or PI^+^ cells were determined. Specific lysis was calculated as described above.

### 2.3 NSCLC mouse model and treatment

All the actions will abide by Regulations for the Administration of Affairs Concerning Experimental Animals issued by State Science and Technology Commission, Guidelines on the treatment of experimental animals issued by Ministry of Science and Technology. The animal use protocol listed below has been reviewed and approved by Animal Ethics Committee of Guangxi University of Chinese Medicine (approval number GXTCMU-EC KS20250000-624). A total of 75 specific pathogen-free (SPF) male C57BL/6 mice (8 weeks old, weighing 19–21 g) were purchased from Changsha Tianqin Biotechnology Co., Ltd. The mice were housed under controlled environmental conditions: room temperature of 25 °C ± 2 °C, relative humidity of 45%–55%, and a 12-h light/dark circadian rhythm, with free access to standard chow and drinking water. Prior to the experiment, the mice were acclimatized to the housing environment for 5–7 days. Subcutaneous tumor-bearing mouse models were established by inoculating LLC cells into the dorsal region of the right hind limb of SPF male C57BL/6 mice (Figure 1A) (19–21). Briefly, each mouse was immobilized on an operating platform, and the hair surrounding the dorsal right hind limb was shaved. After disinfection of the exposed skin with 75% ethanol, a sterile syringe was inserted obliquely into the subcutaneous tissue to form a small circular bleb, followed by injection of the cell suspension. The injection site was pressed gently prior to needle withdrawal. For the other groups, the LLC cell suspension was thoroughly mixed, and 0.1 mL of the suspension was administered per mouse. Tumor growth was monitored by measuring the longest (length) and shortest (width) diameters of the tumors using a vernier caliper. Tumor volume (mm^3^) was calculated using the formula: Volume = (width^2^ × length) × 0.5 (22).

**FIGURE 1:**
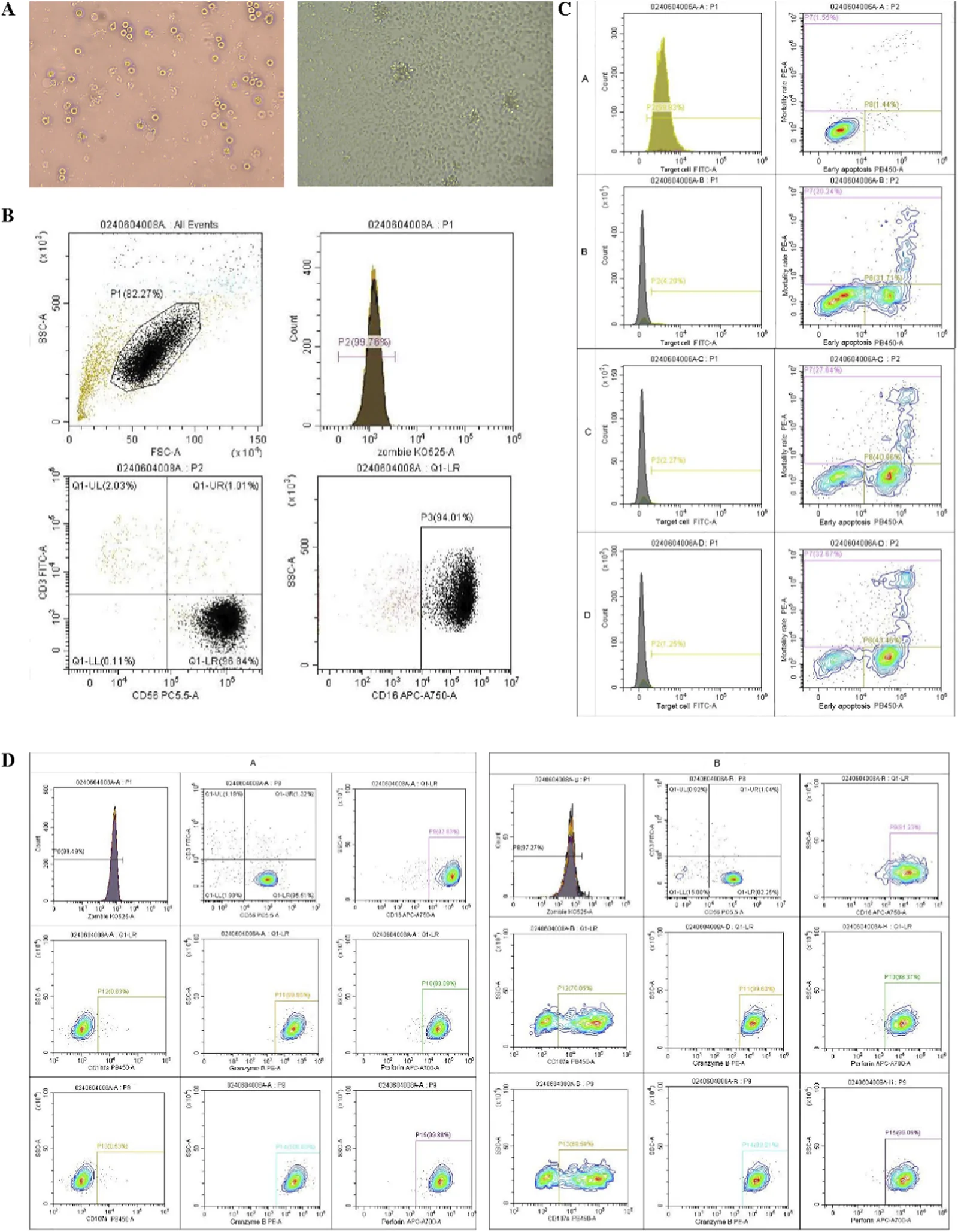
Characterization of ex vivo-expanded human NK cells. **(A)** NK cell morphology on day 1 and day 18 **(B)** Phenotypic identification of NK immune cells **(C)** NK cell function testing **(D)** Kill function testing.

To determine the MED of RT, tumor-bearing mice were randomly divided into five groups (n = 5): the Control group, G1 group, G2 group, G3 group, and G4 group. Mice in the Control group received no irradiation, while those in the G1, G2, G3, and G4 groups were subjected to a single fraction of RT at doses of 10 Gy, 12 Gy, 14 Gy, and 16 Gy, respectively (Figure 1A) (23). Tumor size and body weight were measured on days 0, 3, and 5. Subsequently, all mice were anesthetized with 3.5%–4.0% isoflurane inhalation and sacrificed via cervical dislocation. Tumors were completely excised, immediately snap-frozen in liquid nitrogen, and stored for subsequent analyses.

To determine the MED of NK cell therapy, tumor-bearing mice were randomly divided into five groups (n = 5): the Control group, G1 group, G2 group, G3 group, and G4 group. Mice in the Control group were intravenously injected with an equal volume of PBS, while those in the G1, G2, G3, and G4 groups received a single intravenous injection of NK cells via the tail vein at doses of 2 × 10^6^, 5 × 10^6^, 1 × 10^7^, and 2 × 10^7^ cells per mouse, respectively (Figure 1A) (24, 25). Tumor size and body weight were measured on days 0, 3, and 5. Subsequently, all mice were anesthetized with 3.5%–4.0% isoflurane inhalation and sacrificed via cervical dislocation. Tumor tissues were excised, immediately snap-frozen in liquid nitrogen, and stored for subsequent analyses.

To evaluate the therapeutic efficacy of different treatment regimens, tumor-bearing mice were randomly divided into five groups (n = 5): the Control group, RT group, NK group, RN group, and RN2 group. Mice assigned to the Control group received no RT and were intravenously administered an equal volume of PBS. Animals in the RT group were subjected to a single fraction of RT at a dose of 10 Gy. For the NK group, mice were intravenously injected with a single dose of 2 × 10^6^ NK cells via the tail vein. The RN group underwent a single 10 Gy RT fraction, which was followed by an intravenous injection of 2 × 10^6^ NK cells via the tail vein on the subsequent day. Tumor size was recorded after treatment initiation. To further assess therapeutic efficacy, mice in the RN2 group underwent a second round of treatment: a single 10 Gy RT fraction, followed by an intravenous injection of 2 × 10^6^ NK cells via the tail vein on the subsequent day (Figure 2A). Tumor volume and changes in tumor volume were measured on days 0, 3, and 5. When the tumor volume reached 2000 mm^3^, all mice were anesthetized with 3.5%–4.0% isoflurane inhalation and euthanized via cervical dislocation.

**FIGURE 2:**
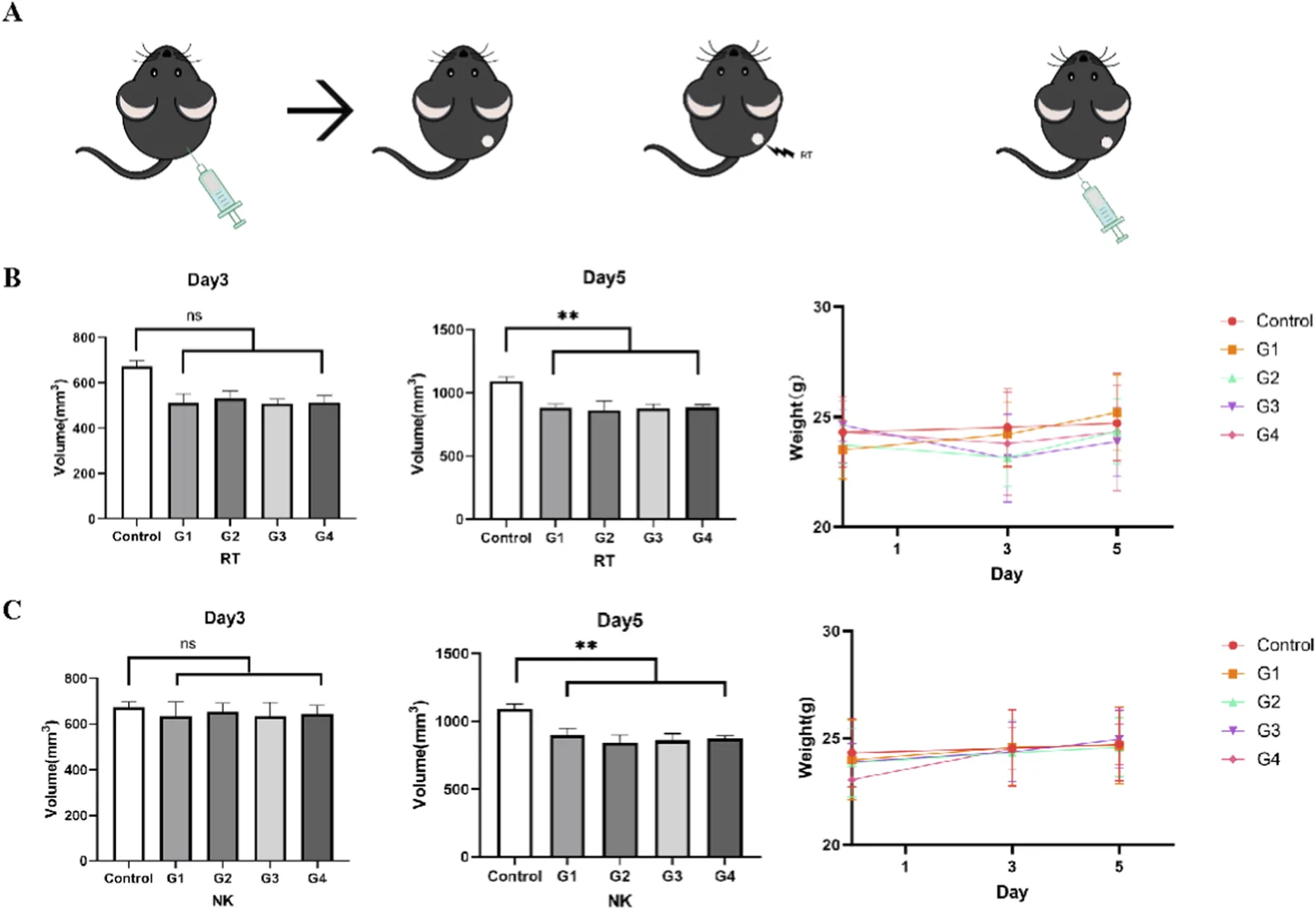
Determination of the MED for RT and NK cell monotherapy. **(A)** Schematic illustration of the establishment of subcutaneous tumor-bearing models via inoculation of LLC cells into the right hind limb of C57BL/6 mice, followed by local RT administration to tumor-bearing mice and NK cell delivery via tail vein injection. **(B)** Evaluation of the efficacy and safety of RT monotherapy. Mice in the Control group received no irradiation, while those in the G1, G2, G3, and G4 groups were subjected to a single fraction of local RT at doses of 10 Gy, 12 Gy, 14 Gy, and 16 Gy, respectively. Changes in tumor volume and mouse body weight were recorded on day 3 and day 5 after RT administration at different doses. **(C)** Evaluation of the efficacy and safety of NK cell monotherapy. Mice in the Control group were intravenously injected with an equal volume of PBS, whereas mice in the NK1, NK2, NK3, and NK4 groups received a single tail vein injection of NK cells at doses of 2 × 10^6^, 5 × 10^6^, 1 × 10^7^, and 2 × 10^7^ cells, respectively. Changes in tumor volume and mouse body weight were measured on day 3 and day 5 following NK cell infusion at different doses. Data are presented as the mean ± SD. **P < 0.05.

### 2.4 Proteomic analysis

Tumor tissues were harvested for proteomic analysis. In the present study, a Thermo Scientific Q Exactive HF (Orbitrap Fusion Lumos) mass spectrometer was employed to perform data-independent acquisition (DIA) based proteomic profiling of tumor samples (26). The raw mass spectrometry (MS) data files were subjected to database searching and parsing. Subsequently, rigorous quality assessment of the MS data was conducted, encompassing quality control (QC) metrics such as peptide sequence matching (PSM) scores and protein identification false discovery rate (FDR), to ensure high reliability of subsequent analyses. Systematic functional annotation was performed for all quantified proteins, including GO annotation and KEGG pathway annotation, to comprehensively delineate the potential functional characteristics and classification information of proteins in the samples. Differential expression analysis of proteins was carried out between comparison groups to screen for DEPs with significant expression alterations. Furthermore, GO functional enrichment analysis and KEGG pathway enrichment analysis were performed on the DEP set to identify significantly enriched biological processes (BPs), molecular functions (MFs), cellular components (CCs), as well as signal transduction and metabolic pathways.

### 2.5 Statistical analysis

All experimental data were analyzed using GraphPad Prism software (version 10.1.2). Data are presented as mean ± standard deviation (SD) unless otherwise specified. Only experiments with three or more biological replicates (n ≥ 3) were included in the statistical analysis. Comparisons among multiple groups were evaluated using one-way analysis of variance followed by appropriate *post hoc* tests for multiple comparisons. P value <0.05 was considered statistically significant.

## 3 Results

### 3.1 Characterization of expanded human NK cells

NK cells expanded *in vitro* from PBMCs exhibited a typical large granular lymphocyte morphology (Figure 1A). Flow cytometric phenotypic analysis revealed that the proportion of CD3^−^CD56^+^ cells reached 96.84%, while CD3^−^CD16^+^CD56^+^ cells accounted for 91.04% (Figure 1B). Functional assays demonstrated that, in the unstimulated state, NK cells constitutively expressed high levels of granzyme B (99.95%) and perforin (99.09%), whereas the expression of CD107a was extremely low (0.63%). Following stimulation with K562 cells, the expression of CD107a was significantly upregulated to 70.85%, while granzyme B and perforin maintained high expression levels (99.63% and 98.37%, respectively). These findings indicated that the expanded NK cells possessed robust degranulation potential and cytotoxic capacity (Figure 1C). *In vitro* cytotoxicity assays further showed that the killing effect of NK cells on target cells was dose-dependently enhanced under different E:T ratios. Specifically, at an E:T ratio of 5:1, the early apoptotic rate and total killing rate were 30.27% and 48.96%, respectively; these values increased to 39.52% and 65.61% at an E:T ratio of 10:1, and reached the highest levels of 42.02% and 73.14% at an E:T ratio of 20:1 (Figure 1D). Collectively, these results confirmed that the expanded NK cells displayed a canonical phenotypic profile and exerted significant specific and dose-dependent cytotoxic activity against target cells.

### 3.2 Determination of MEDs

To determine whether RT combined with NK cell immunotherapy exerts superior therapeutic efficacy compared to monotherapy in NSCLC, we first established a subcutaneous LLC cell xenograft model in C57BL/6 mice, followed by systematic evaluation of the efficacy and safety of the combination strategy (Figure 2). To minimize radiation-induced damage to normal tissues, local irradiation was delivered to tumors implanted in the right hind limb of mice, with four dose groups set, 10, 12, 14, and 16 Gy. For the NK cell treatment groups, different doses of NK cells, 2 × 10^6^, 5 × 10^6^, 1 × 10^7^, and 2 × 10^7^ cells were administered via tail vein injection. Tumor volumes were recorded on days 0, 3, and 5 post-treatment, and changes in mouse body weight were monitored concurrently to assess treatment-related toxicity.

The results showed that on day 3 post-RT, there were no significant differences in tumor volume between each RT dose group and the Control group. By day 5, the RT groups exhibited a significant tumor suppressive effect (P < 0.05); however, no statistically significant differences were observed among the different RT dose groups (Figure 2B). Considering that the 10 Gy dose was sufficient to achieve significant tumor growth inhibition and no obvious toxic reactions were observed in any mice, 10 Gy was determined as the MED for RT. Regarding NK cell treatment, similar to the RT groups, no significant differences in tumor volume were detected between each NK cell dose group and the Control group on day 3. By day 5, the NK cell treatment groups also showed a significant tumor suppressive effect (P < 0.05), but no statistical differences existed among the various NK cell dose groups (Figure 2C). Given that the 2 × 10^6^ cells dose could effectively inhibit tumor growth without inducing obvious adverse reactions, this dose was defined as the MED for NK cell therapy. Notably, neither treatment induced significant toxic side effects at the tested doses.

### 3.3 Effect of RT and NK cell combination

To clarify whether RT combined with NK cell immunotherapy confers greater advantages over monotherapy in NSCLC, we established a subcutaneous LLC cell xenograft model in C57BL/6 mice and systematically evaluated the efficacy and adverse reaction of this combination regimen (Figure 3). The results demonstrated that on day 3 post-treatment, there were no significant differences in tumor volume between each treatment group and the Control group. By day 5, compared with the Control group, the RN group exhibited the most significant tumor growth inhibitory effect (P < 0.05), indicating that this combination strategy possessed potent antitumor activity *in vivo* (Figure 3B). In contrast, although the RT monotherapy group or NK monotherapy group induced a certain degree of tumor volume reduction, the differences between these two groups were not statistically significant, suggesting that the tumor suppressive effect of monotherapy was limited (Figure 3B). Notably, no obvious toxic or side effects were observed in the combined treatment group throughout the experimental period, which initially confirmed its favorable safety profile (Figure 3B). Furthermore, the tumor volume in the RN group was significantly smaller than that in the RT group and NK group (P < 0.05), further verifying that combined treatment could inhibit the growth of LLC tumors in mice more effectively than monotherapy. To investigate the sustained effect of the treatment, a second round of combined treatment was administered to the RN2 group. The results showed that compared with the RN group receiving a single round of combined treatment, the tumor growth in the RN2 was further slowed down, and no obvious toxic reactions were still observed (P < 0.05) (Figure 3C).

**FIGURE 3:**
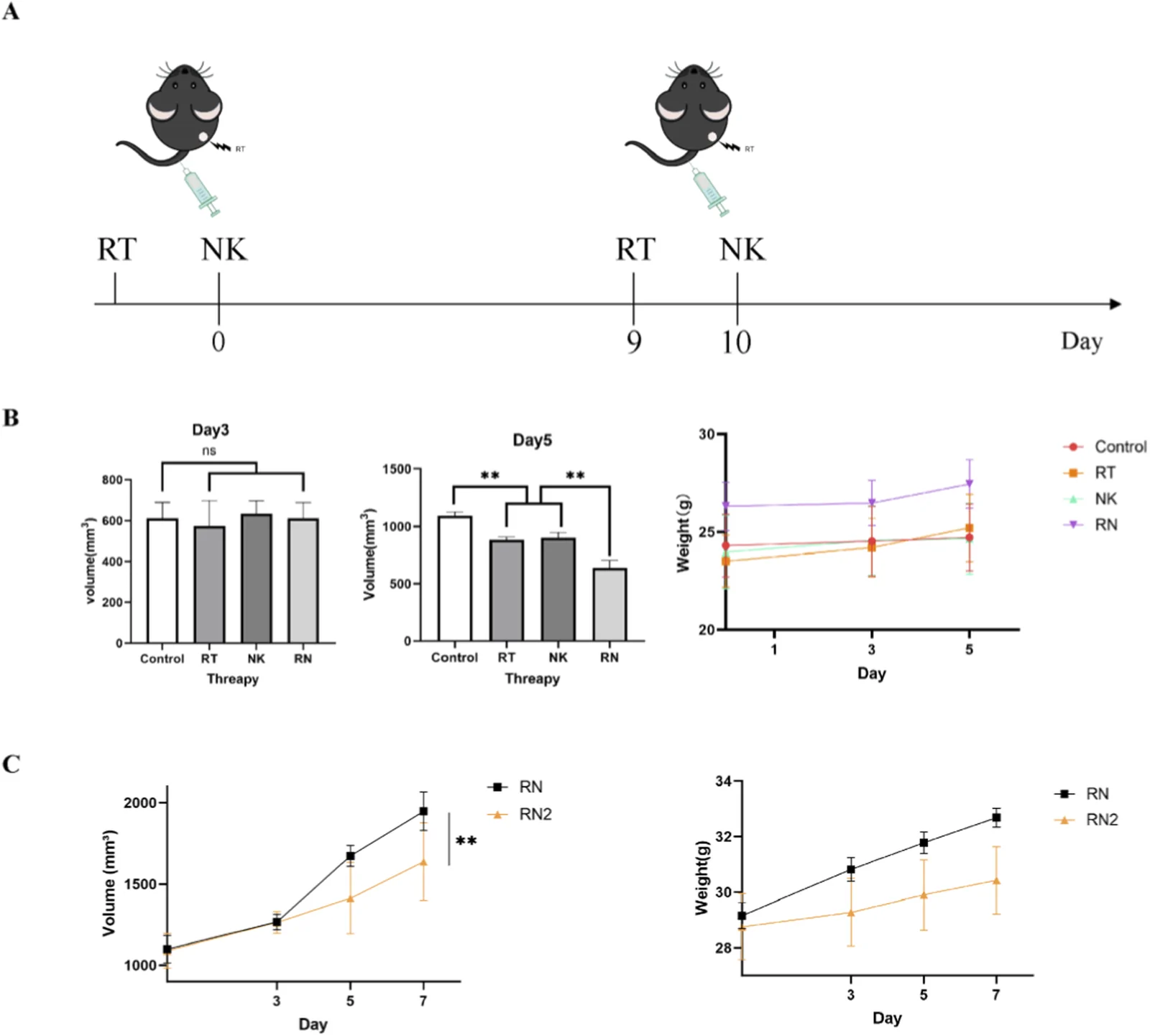
Synergistic anti-tumor effect of RT combined with NK cell therapy. **(A)** Schematic diagram of the combined treatment regimen: tumor-bearing mice received two rounds of RT combined with NK cell therapy intervention. **(B)** Efficacy and safety of single-round combined treatment. **(C)** Efficacy and safety of two-round combined treatment. Changes in tumor volume and body weight of mice in each group were recorded on day 3 and day 5 post-treatment. Mice in the Control group received no RT and were intravenously injected with PBS; those in the RT group were subjected to a single fraction of 10 Gy irradiation and were intravenously injected with PBS; mice in the NK group were administered 2 × 10^6^ NK cells via tail vein injection; and animals in the RN group received a single 10 Gy irradiation followed by tail vein injection of 2 × 10^6^ NK cells on the next day. Mice in the RN2 group were given a second round of 10 Gy irradiation on day 9, with tail vein injection of 2 × 10^6^ NK cells on the subsequent day. **P < 0.05.

### 3.4 Proteomic profiling and quality control

To investigate RT combined with NK cell immunotherapy for NSCLC, mouse tumor samples were collected for proteomic sequencing. DIA was employed for MS data acquisition. Briefly, the entire MS scanning range was divided into several windows based on m/z; all precursor ions within each window were then fragmented and detected, and fragment ion information of all precursor ions was collected for protein qualitative and quantitative analysis. A total of 8211 proteins were quantified via DIA-based proteomics. Figure 4A shows the distribution of protein expression abundances across all samples, intuitively revealing differences in protein expression patterns among different samples, with distinct clustering characteristics of protein expression abundances in samples from different groups. The expression trends of all proteins were classified into 9 clusters, and the normalized expression abundance of each cluster exhibited a unique variation pattern among the Control group, RT group, and NK group, reflecting the heterogeneity of protein expression trends (Figure 4B). Further analysis indicated that each module displayed different upregulation or downregulation trends among the Control group, RT group, and NK group. Combined with the clustering tree, the consistency of protein expression within each module was observed, and there were significant differences in the expression trends of different modules among the groups, reflecting the specific regulation of protein expression modules by group (Figure 4C). In principal component analysis (PCA), the contribution rate of PCA1 was 25.02% and that of PCA2 was 16.06%. The scatter plot showed that samples from the Control group, RT group, and NK group exhibited obvious clustering in the PCA space, indicating significant differences in protein expression profiles among the groups, and PCA could effectively distinguish samples from different groups (Figure 4D).

**FIGURE 4:**
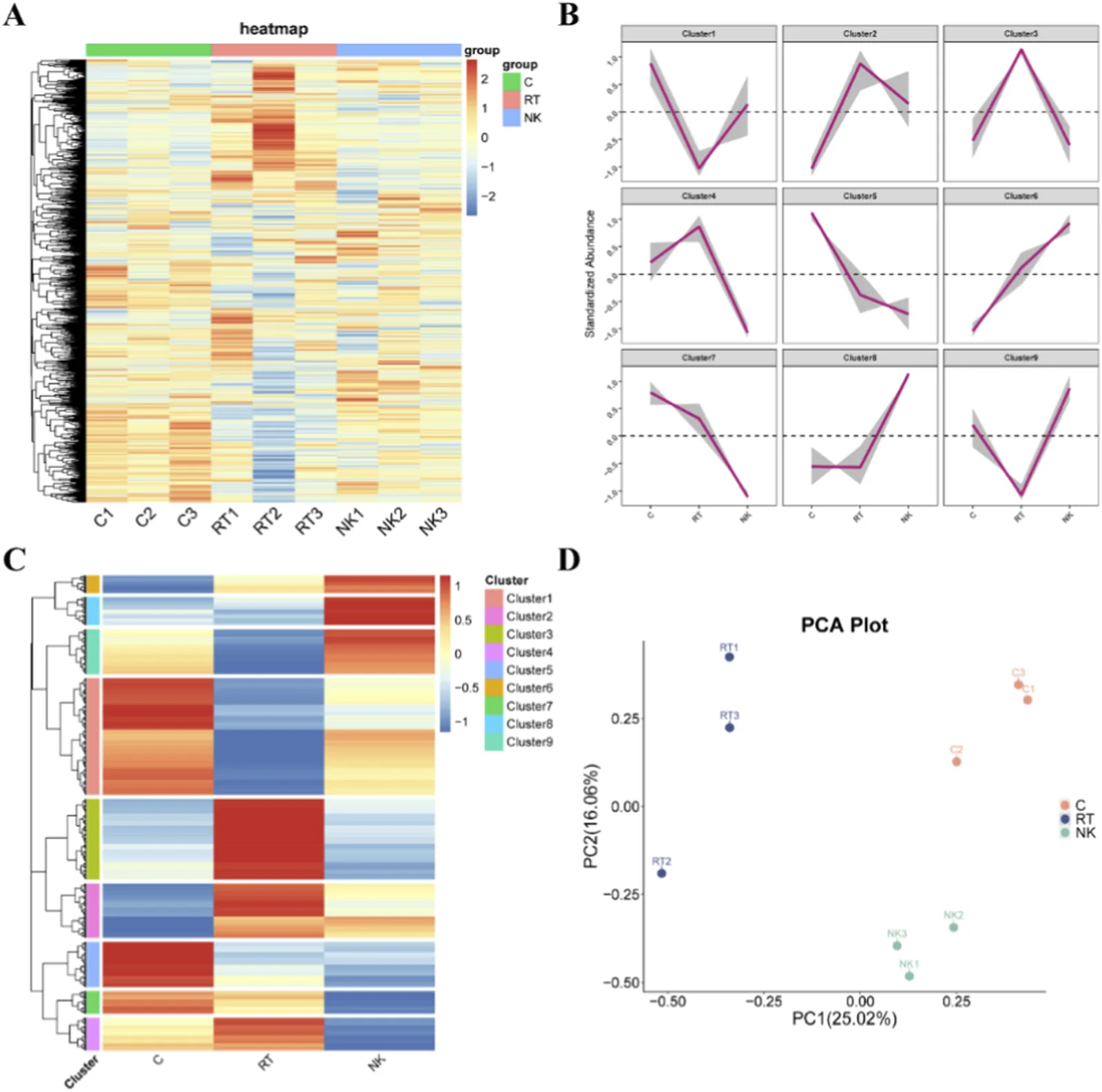
QC and overview of the proteomic dataset. **(A)** Distribution of protein expression abundances across all samples. Red indicates high expression, and blue indicates low expression; the clustering tree is also shown. **(B)** Expression trend plots of all groups generated based on protein quantification results. All protein expression trends were classified into 9 clusters, and the expression trend of each cluster is presented. **(C)** Cluster heatmap of all sample groups and proteins constructed based on protein quantification results. Different columns in the figure represent different group names, and different rows represent different proteins. The color intensity indicates the quantitative level of proteins in each group. **(D)** Description of samples by PCA. The abscissa represents the first principal component, and the percentage in parentheses indicates the contribution rate of the first principal component to sample differences; the ordinate represents the second principal component, and the percentage in parentheses indicates the contribution rate of the second principal component to sample differences. Each point in the figure represents an individual sample, and different colors indicate that the samples belong to different groups.

### 3.5 Proteomic alterations induced by RT

To identify DEPs, Student’s t-test was employed to analyze the relative quantitative values of each protein between comparison groups, and the corresponding p-values were calculated. The criteria for defining upregulated DEPs were Fold Change >1.2 and p < 0.05, while downregulated DEPs were defined as Fold Change <0.833 and p < 0.05. The upregulated and downregulated proteins were combined to obtain the total DEP set.

Compared with the Control group, a total of 281 significantly upregulated proteins and 628 significantly downregulated proteins were identified in the RT group (Figures 5A–C). Based on proteomic analysis, GO biological process enrichment analysis revealed that the DEPs in the RT group were significantly enriched in pathways such as DNA replication, DNA replication initiation, and mismatch repair (Figure 5D). These results indicated that while RT induces extensive DNA damage, it may specifically activate or disrupt the operation of the DNA replication machinery and the function of related repair systems in cancer cells. The significant enrichment of these processes suggests that tumors may adapt to damage and maintain survival after RT by regulating replication fork stability, restarting stalled replication processes, or altering DNA repair fidelity. This provides important clues for in-depth elucidation of RT resistance mechanisms and the discovery of potential synergistic therapeutic targets.

**FIGURE 5:**
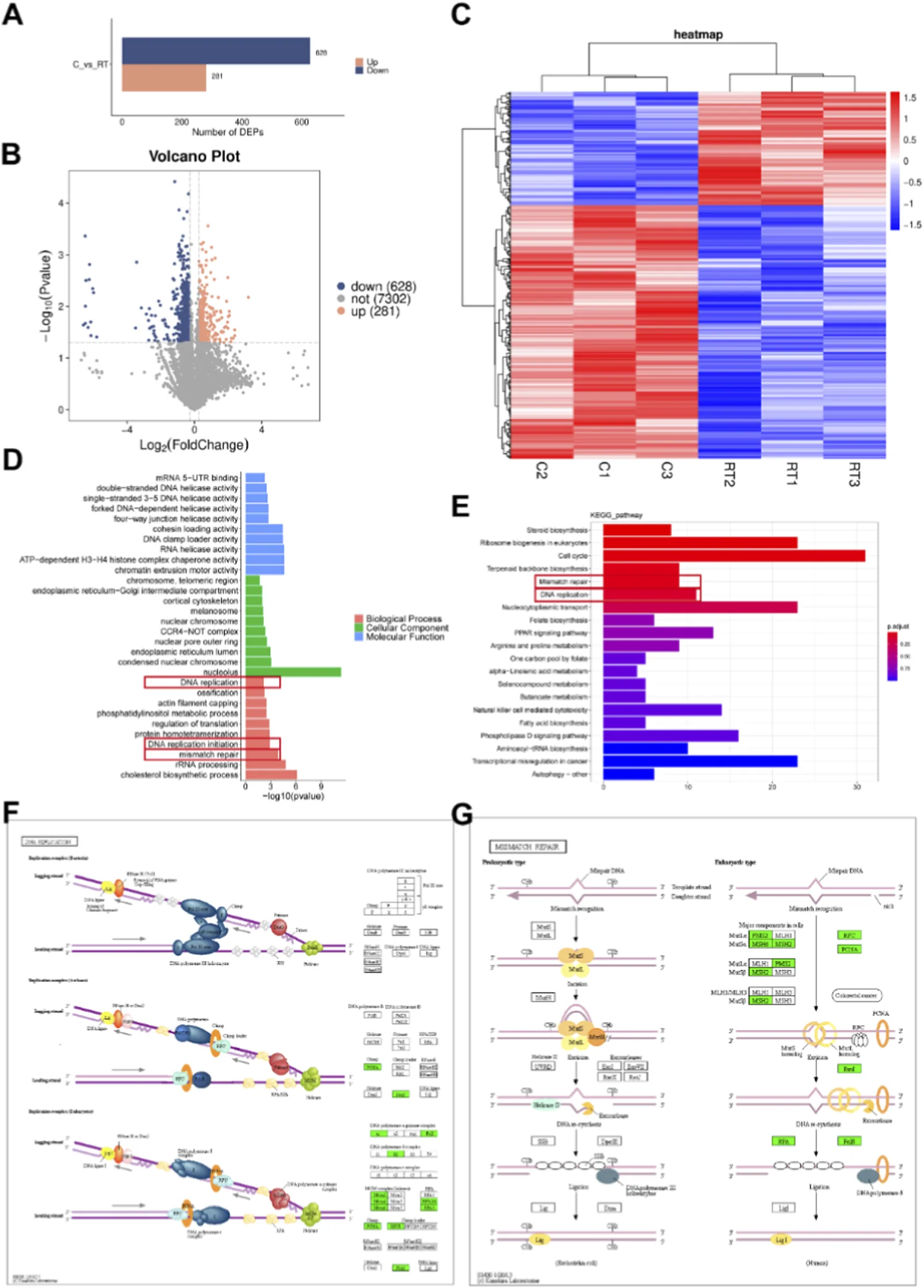
Proteomic profiling of tumor tissue following RT. **(A)** Number of DEPs. **(B)** Volcano plot of DEPs. **(C)** Cluster heatmap of DEPs. Different columns in the figure represent different samples, and different rows represent different proteins. The color intensity indicates the expression level of proteins in the samples. **(D)** GO enrichment analysis of DEPs. **(E)** KEGG enrichment analysis of DEPs. **(F)** DNA replication pathway map. **(G)** Mismatch repair pathway map. Blue dots represent downregulated DEPs, orange-red dots represent upregulated DEPs, and gray dots represent non-DEPs.

To further clarify the molecular mechanisms involved in the DEPs, KEGG pathway enrichment analysis was performed. The results were highly consistent with the GO analysis, showing that the DEPs were significantly enriched in multiple DNA metabolism-related pathways (Figure 5E), among which the DNA replication and mismatch repair pathways were particularly prominent (Figures 5F,G). These results further confirmed, through an annotation system based on known molecular interaction networks, that RT specifically disrupts the core regulatory network of DNA synthesis and repair in tumor cells.

### 3.6 Proteomic alterations induced by NK cell therapy

Compared with the Control group, a total of 236 significantly upregulated proteins and 249 significantly downregulated proteins were identified in the NK group, with no significant changes in the expression of another 7,726 proteins (Figures 6A–C). GO functional enrichment analysis based on DEPs showed that protein changes after NK cell therapy were significantly concentrated in BPs such as complement activation and its classical pathway (Figure 6D). These results suggest that NK cell therapy may be associated with the regulation of complement cascade components, a crucial innate immune effector system of the body. The significant activation of the classical complement pathway suggested that after the interaction between NK cells and tumor cells, NK cells may induce the formation of antigen-antibody complexes on the surface of target cells or directly alter the characteristics of tumor cell membranes, thereby initiating the classical complement cascade centered on Complement Component 1q (C1q) recognition.

**FIGURE 6:**
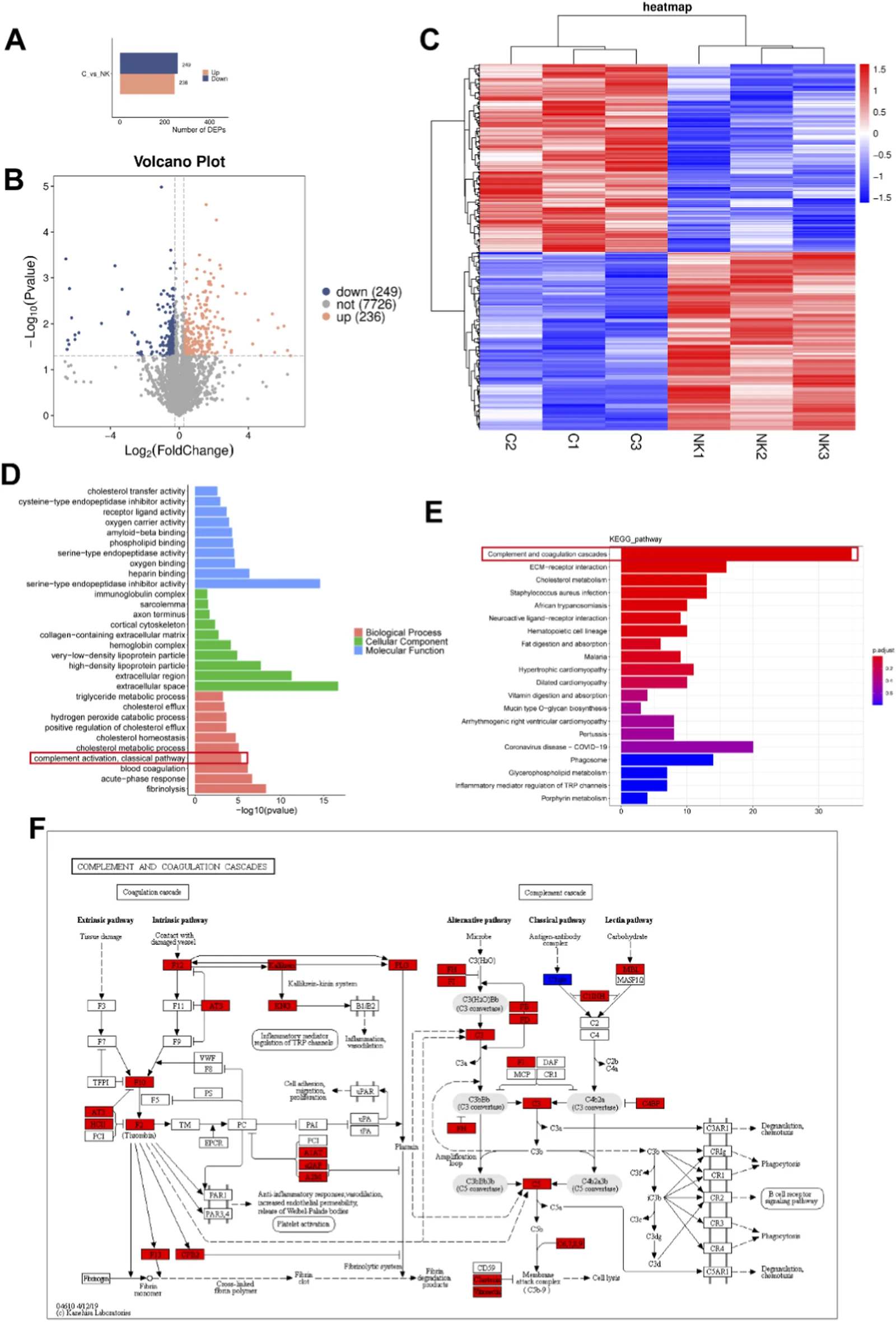
Proteomic profiling of tumor tissue following NK cell therapy. **(A)** Number of DEPs. **(B)** Volcano plot of DEPs. Blue dots represent downregulated DEPs, orange-red dots represent upregulated DEPs, and gray dots represent non-DEPs. **(C)** Cluster heatmap of DEPs. Different columns in the figure represent different samples, and different rows represent different proteins. The color intensity indicates the expression level of proteins in the samples. **(D)** GO enrichment analysis of DEPs. **(E)** KEGG enrichment analysis of DEPs. **(F)** Complement and coagulation cascades pathway map.

KEGG pathway enrichment analysis further supported the above conclusion, showing that DEPs were significantly enriched in the “complement and coagulation cascades” pathway (Figure 6E). The pathway mapping diagram clearly revealed that this enrichment was mainly driven by the upregulated expression of a series of key proteins in the classical complement pathway (Figure 6F), specifically including C1q subunits responsible for initial recognition, C2 and C4 components involved in C3 convertase formation, and effector proteins such as C5, C8, and C9 that constitute MACs. This complete set of protein expression changes covering the initiation to effector stages is consistent with the hypothesis that NK cell therapy may activate the classical complement pathway.

## 4 Discussion

In recent years, immunotherapy has emerged as a crucial first-line therapeutic strategy for NSCLC. A growing body of evidence indicates that immunotherapy and RT exert significant synergistic effects in local and distant tumor control (27). In the exploration of combined therapeutic modalities involving first-line chemotherapy, immunotherapy, and RT for NSCLC patients, the determination of optimal radiation dose is of paramount importance (7, 28). With the continuous advancement of radiation therapy technologies, clinical treatment modalities have become increasingly diversified, mainly including stereotactic body radiation therapy (SBRT) and conventional fractionated RT (29). Although SBRT and conventional fractionated RT differ in radiobiological characteristics, cell-killing mechanisms, and clinical indications, both exert antitumor effects through ionizing radiation and thus fall within the scope of RT (27, 30, 31). Therefore, in combined treatment regimens, individualized selection and optimization of RT modalities and dose parameters should be performed based on factors such as tumor characteristics, therapeutic goals, and safety profiles, to maximize their synergistic antitumor effects with immunotherapy (32, 33). Our study demonstrates that combining RT with human NK cell therapy induces a synergistic anti-tumor effect in an NSCLC model, superior to either modality alone.

The results of this study demonstrated that combined RT and NK cell therapy exerted a significant synergistic antitumor effect in LLC xenograft model in C57BL/6 mice. *In vivo* efficacy data revealed that the combined treatment was superior to either monotherapy in both inhibiting tumor growth and prolonging survival time. Furthermore, it exhibited favorable safety profiles within the tested dose range, with no obvious toxic or side effects observed. Notably, no significant differences in tumor volume were detected between each treatment group and the Control group on day 3 post-treatment, indicating that the effects of both RT-induced direct cytotoxicity and NK cell-mediated direct killing on tumor volume were delayed to a certain extent. By day 5, although the monotherapy groups showed a trend of tumor suppression, the effect was limited and no significant differences were observed between the groups; in contrast, the combined treatment group had already exhibited significantly stronger tumor suppressive capacity at this time point. These kinetic changes indicate that the synergistic efficacy of the combination therapy is not merely a simple superposition of early effects. Instead, it stems from the dynamic interplay and synergistic amplification of distinct mechanisms over time, ultimately achieving a potent cooperative anti-tumor effect *in vivo*. We acknowledge that our study did not include a control group receiving inactivated or dead human NK cells. Such a control would have been valuable to distinguish specific NK cell-mediated antitumor effects from non-specific inflammatory responses triggered by the infusion of xenogeneic cellular material. In the absence of this control, we cannot definitively exclude the possibility that the observed antitumor activity was partially contributed by host immune responses to the xenogeneic cells themselves. Future studies incorporating dead-cell controls or NK cell-depleting antibodies are warranted to clarify the specificity of the observed effects. To further evaluate the sustained effect and tolerability of the combined treatment, a second round of combined treatment was administered to the RN2 group. The results showed that compared with the RN group receiving only one round of treatment, tumor growth was further slowed down was significantly prolonged in the RN2 group. Importantly, even after the second round of treatment, no obvious weight loss, abnormal activity, or other toxic manifestations were observed in any mice, indicating that the combined regimen maintained favorable safety and tolerability while achieving cumulative antitumor effects. This result further supports the potential advantages of RT combined with NK cell therapy in expanding the therapeutic window and maintaining treatment safety. We acknowledge that our safety evaluation was primarily based on body weight monitoring and general clinical observations, which, while informative, do not constitute a comprehensive toxicological assessment. Hematological parameters, serum biochemical markers of organ function, and histopathological examination of major organs were not performed in the current study. Therefore, although we observed no overt signs of systemic toxicity, the possibility of subclinical organ damage or hematological abnormalities cannot be excluded. Future studies incorporating systematic toxicity profiling—including organ histopathology, serum biochemistry, and immune organ indices—will be essential to rigorously evaluate the safety margin of this combination strategy and to support its potential clinical translation.

To elucidate the molecular basis underlying the synergistic anti-tumor effects of radiotherapy combined with NK cell therapy, we performed a systematic proteomic analysis of tumor samples from each group. Our findings suggest that this synergy stems from the spatiotemporal coupling of two distinct biological processes: the DNA damage response network triggered by radiotherapy and the innate immune regulatory network activated by NK cells. Proteomic data indicated that radiotherapy induced significant DNA double-strand breaks in tumor cells, thereby activating stress pathways centered on the DNA damage response. The activation of these pathways not only mediated cell cycle arrest and apoptosis but, more critically, elicited DNA replication stress and disrupted the homeostasis of the mismatch repair system (28). These alterations may have established a foundation for enhancing the subsequent sensitivity of tumor cells to immune attacks. Concurrently, proteomic profiling clearly identified NK cell infiltration and activation as pivotal driving events in triggering the innate immune response. We hypothesize that the complement system serves as a critical mediator in the combination regimen. Proteomic profiling indicated a broad activation of complement branches, particularly evidenced by the substantial upregulation of key constituents in the classical and lectin pathways. Synthesizing our proteomic findings with established literature, we propose a multi-level, multi-target synergistic mechanism model (29, 34, 35). Radiotherapy acts as the initial trigger; by inducing DNA damage and replication stress, it not only directly inhibits tumor growth but, more critically, may alter the phenotypic characteristics of tumor cells and the state of the tumor microenvironment. Specifically, radiotherapy-pretreated tumor cells likely upregulate ligands for NK cell-activating receptors and release inflammatory cytokines, thereby establishing a favorable landscape for NK cell homing and activation. Within this context, activated NK cells serve as a pivotal bridge in the combination regimen. On one hand, they exert direct cytotoxicity through the release of perforin and granzymes, causing physical membrane damage. On the other hand, they may remodel the local immune microenvironment via the secretion of cytokines such as IFN-γ (29). This dual function not only damages tumor cells but also ‘mobilizes’ the host complement system—likely through the exposure of conserved complement-activating signals like phosphatidylserine—converting it into an effective anti-tumor effector. Upon complement activation, the cleavage product C5a acts as a potent chemoattractant, recruiting additional innate immune cells (e.g., neutrophils and macrophages) to the tumor site and establishing a positive inflammatory feedback loop (34). Concurrently, the formation of the membrane attack complex (MAC) creates pores in the tumor cell membrane, directly mediating osmotic lysis. Ultimately, MAC-mediated membrane damage, NK cell cytotoxicity, and radiotherapy-induced DNA damage converge on tumor cells simultaneously, constituting a multi-lethal strike from both the intracellular and surface levels (35). This multi-layered attack strategy—spanning from nuclear events to membrane disruption and from direct killing to immune mobilization—likely underpins the “1 + 1>2” synergy observed in the combination therapy. This model integrates radiotherapy-induced DNA damage, NK cell cytotoxicity and immunomodulation, and the amplifying effects of the complement system into a cohesive framework. However, we acknowledge several important caveats and alternative interpretations that warrant consideration. First, the proteomic changes observed in the monotherapy groups may represent compensatory or stress-responsive signatures rather than direct on-target engagement of the therapeutic agents. For instance, upregulation of certain stress-related proteins (e.g., heat shock proteins or antioxidant enzymes) could reflect cellular adaptation to injury rather than specific pathway modulation that is directly relevant to the combination effect. Second, in the absence of proteomic data from the combination group, we cannot distinguish whether the functional synergy arises from true signaling crosstalk between the DNA damage and immune pathways, from pharmacokinetic or pharmacodynamic interactions (e.g., radiotherapy-induced vascular permeability changes that enhance NK cell infiltration, or altered drug metabolism), or from bystander activation of the murine endogenous immune system in response to xenograft rejection or RT-induced tumor cell debris. Our current experimental design does not allow us to definitively differentiate between these non-mutually exclusive possibilities. Third, while our kinetic observation of significant antitumor effect as early as Day 5 post-infusion is suggestive of human NK cell activity prior to xenograft rejection, it does not formally exclude a contribution from host murine immune cells responding to the treatment. Therefore, while our integrative analysis of monotherapy proteomes provides a useful hypothesis-generating framework for future mechanistic dissection, we emphasize that the precise molecular events underlying the combination therapy—and particularly the identity of synergistic signaling nodes unique to the dual treatment—remain to be definitively established. Future studies employing complement blockade, NK cell depletion models, pathway-specific inhibitors, or CRISPR-based gene editing in appropriate humanized mouse models are warranted to functionally validate this model and further elucidate the specific contributions of each component to the synergistic efficacy.

In this study, human-derived NK cells were infused into immunocompetent C57BL/6 mice bearing murine LLC tumors, which confers a unique value in hypothesis generation in the context of mechanistic exploration. Radiotherapy may create a favorable microenvironment that potentially facilitates NK cell recruitment. By combining *in vivo* experiments with proteomic analysis, we systematically evaluated the antitumor efficacy and potential molecular correlates of local RT combined with systemic NK cell adoptive transfer. NK cell therapy was associated with activation of complement pathway components, suggesting a potential interplay between NK cells and the complement system that warrants further functional investigation. Conventionally, in preclinical studies investigating NK cell-based immunotherapies, human-derived tumor models are employed, wherein human NK cells are administered to evaluate their antitumor efficacy (21, 36). This model introduces a novel dimension of complexity, namely the attack of murine tumor cells by human immune effector cells. This design goes beyond traditional allogeneic or xenograft models, facilitating the elucidation of the role of cross-species immune recognition in combined therapy. In this model, the interaction between infused NK cells and tumors is neither restricted by MHC antigens nor supported by co-evolved precise recognition pairs (36). Nevertheless, the combined treatment still exhibited significant synergistic effects, raising a core scientific question: How does RT enable human NK cells to effectively kill murine tumors independent of classical MHC-restricted recognition? Our data suggest that RT-induced global remodeling of the TIME and the triggered endogenous innate immune cascade play pivotal roles in this process (37). RT-induced extensive cell death, vascular damage, and stromal remodeling release a large number of DAMPs, creating a strong “danger signal” milieu within the tumor (38, 39). This environment non-specifically activates and recruits the host’s own innate immune cells (especially macrophages and neutrophils), leading to a significant increase in local inflammatory cytokine levels (37). Circulating human NK cells may be pre-activated by systemic inflammatory cytokines. More importantly, when recruited to this highly inflammatory tumor site, their functional status is strongly driven by high local concentrations of cytokines, thereby entering a hyperactivated, cytokine-dependent cytotoxic mode. At this stage, their recognition of target cells may rely more on the interaction between activating receptors and constitutively expressed adhesion molecules on the tumor cell surface, rather than the classical MHC-I deficiency recognition mechanism (40). A critical caveat of this study is the cross-species design using human NK cells in an immunocompetent mouse model. While we observed significant tumor inhibition as early as Day 5 post-infusion—a time point preceding the typical window of xenograft rejection (6–12 days) —we acknowledge that this indirect kinetic evidence does not replace direct *in vivo* tracking of human NK cell persistence (41). In summary, through an innovative cross-species therapeutic model, this study reveals that the deep synergistic mechanism of RT combined with human NK cell therapy for NSCLC may not primarily depend on classical direct tumor recognition, but rather originates from a cascading amplified innate immune response, RT shapes a “dangerous” inflammatory TIME; this environment recruits and strongly activates human NK cells; activated NK cells, through their cytotoxic effects, “translate” and “amplify” RT-induced damage signals into conserved danger signals widely recognizable by the host complement system, thereby triggering a potent bystander killing effect independent of specific recognition. This finding broadens our understanding of the synergistic mechanism between RT and immunotherapy, and establishes the innate immune system (especially the complement pathway) as an indispensable key effector component and potentiation target in combined therapy. It provides a new theoretical basis and direction for the future development of more effective combined immunotherapeutic strategies for solid tumors.

Unlike T cells, NK cells can directly bind to tumor cells and induce cytotoxicity without the requirement for tumor-specific antigen recognition or the risk of graft-versus-host disease (42). However, NK cell-based immunotherapy has shown limited efficacy in solid tumors, largely due to poor infiltration of lymphocytes and NK cells within the TIME, which is associated with unfavorable prognosis in solid cancers (43–46). Radiotherapy has been reported to facilitate the effective infiltration and activation of NK cells into the TIME, thereby enhancing their interaction with tumor cells and mediating efficient cytotoxicity (17, 47, 48). Thus, the combination of RT and NK cell immunotherapy represents a conceptually promising strategy for solid tumors. In the present study, our proteomic analyses revealed that the combination of RT and NK cell treatment was associated with alterations in immune-related pathways, providing preliminary evidence suggestive of immune-modulatory effects. However, we acknowledge an important limitation: we did not directly characterize tumor-infiltrating immune cell populations or quantify local cytokine production. Consequently, while our proteomic data indicate molecular changes consistent with immune remodeling, they do not directly establish changes in the composition, abundance, or functional state of immune cells within the TIME, nor do they definitively determine whether the combined treatment shifts the local inflammatory or immunosuppressive cytokine milieu. Therefore, our findings should be interpreted as correlative evidence suggesting an association between RT/NK treatment and immune-related remodeling, rather than definitive proof of cellular- or cytokine-level changes within the TIME. Future studies incorporating comprehensive immune cell profiling and localized cytokine measurements will be required to further define the underlying immunological mechanisms. Despite these limitations, our preliminary findings, obtained in an early-stage NSCLC progression model, suggest that the combination of RT and NK cell-based immunotherapy may offer a novel therapeutic approach worthy of further investigation in solid cancers.

## 5 Conclusion

In conclusion, RT combined with NK therapy demonstrated synergistic antitumor efficacy and no overt toxicity was observed, while repeated treatment further delayed tumor progression. Proteomic profiling suggested that RT primarily disrupted DNA replication and repair networks, whereas NK cell therapy was associated with selective activation of the classical complement pathway. However, these findings remain correlative and do not establish a causal or functional relationship between these molecular alterations and the therapeutic effects. Further mechanistic studies are therefore warranted to validate these pathways and elucidate their functional contributions to the antitumor efficacy of RT combined with NK cell therapy.

## Data Availability

The original contributions presented in the study are included in the article/supplementary material, further inquiries can be directed to the corresponding authors.
